# The Prey Pathway: A Regional History of Cattle (*Bos taurus*) and Pig (*Sus scrofa*) Domestication in the Northern Jordan Valley, Israel

**DOI:** 10.1371/journal.pone.0055958

**Published:** 2013-02-06

**Authors:** Nimrod Marom, Guy Bar-Oz

**Affiliations:** Laboratory of Archaeozoology, University of Haifa, Haifa, Israel; University of Oxford, United Kingdom

## Abstract

The faunal assemblage from the 9^th^-8^th^ millennium BP site at Sha'ar Hagolan, Israel, is used to study human interaction with wild suids and cattle in a time period just before the appearance of domesticated animals of these species in the Jordan Valley. Our results, based on demographic and osteometric data, indicate that full domestication of both cattle and suids occurred at the site during the 8^th^ millennium. Importantly, domestication was preceded in both taxa by demographic and metric population parameters indicating severe overhunting. The possible role of overhunting in shaping the characteristics of domesticated animals and the social infrastructure to ownership of herds is then explored.

## Introduction

Understanding of the role of humans as constant modifiers of their ecological niches is reshaping at present our understanding on the beginning of agriculture [1]. The effects of the demographic pulse following the rise of food-producing, settled communities in the 11^th^-10^th^ millennia BP [2], [3] has seen an exponential growth in the area occupied by human-constructed, homogenized agricultural landscapes. We perceive this expansion as axial to the evolving interaction at that time between humans, wild cattle and wild boar that eventually led to the domestication of these species. The emphasis here is on viewing domestication as an unfolding process—*evolution*, rather than sudden event. In this respect we part with older approaches seeking clear cut-off points between domesticated livestock species and their wild progenitors in the region, approaches which are being replaced by more nuanced documentation of the selective pressures introduced in the process of domestication [4].

This documentation involves the decoding of genetic, metric and demographic markers that can be directly linked to an evolving human-animal relationship [5]. The most common markers used to document domestication are demographic, bio-geographic and morphological changes that occurred in the transformation of a wild species into a domesticated one. Its onset can be found in sheep, goat, cattle and pigs at certain parts of the Near East from the 12^th^ millennium BP onwards. Age-at-death and sex ratio analyses of early livestock taxa show increasing departure from the prime-adult pattern that typified Palaeolithic and Epipalaeolithic hunting to selective culling of younger male animals. This pattern can be seen in a wide region stretching from the Taurus-Zagros arc to the Upper Euphrates [6], [7]. It is now commonly believed that prey population management preceded allometric body-size reduction. The recent discoveries of morphologically-wild populations of goats, cattle, suids and even fallow deer in 11^th^ millennium BP Cyprus, brought there by Neolithic colonists, is a striking demonstration of such human involvement [8].

Genetic studies further revealed the occurrence of multiple domestication events for suids [9] and cattle [10]. There is therefore good grounds to the opinion that domestication of cattle and pigs was carried out at multiple times and places within the Near East [4]. Under these conditions, micro-regional historical-biological studies of target species are needed. Such studies should ideally apply both fine-tuned demographic (sex-ratio and mortality profiles), biometric (body-size reduction) and genetic markers to allow detailed exploration of local animal selection in their native habitat under human influence [4].

**Figure 1:**
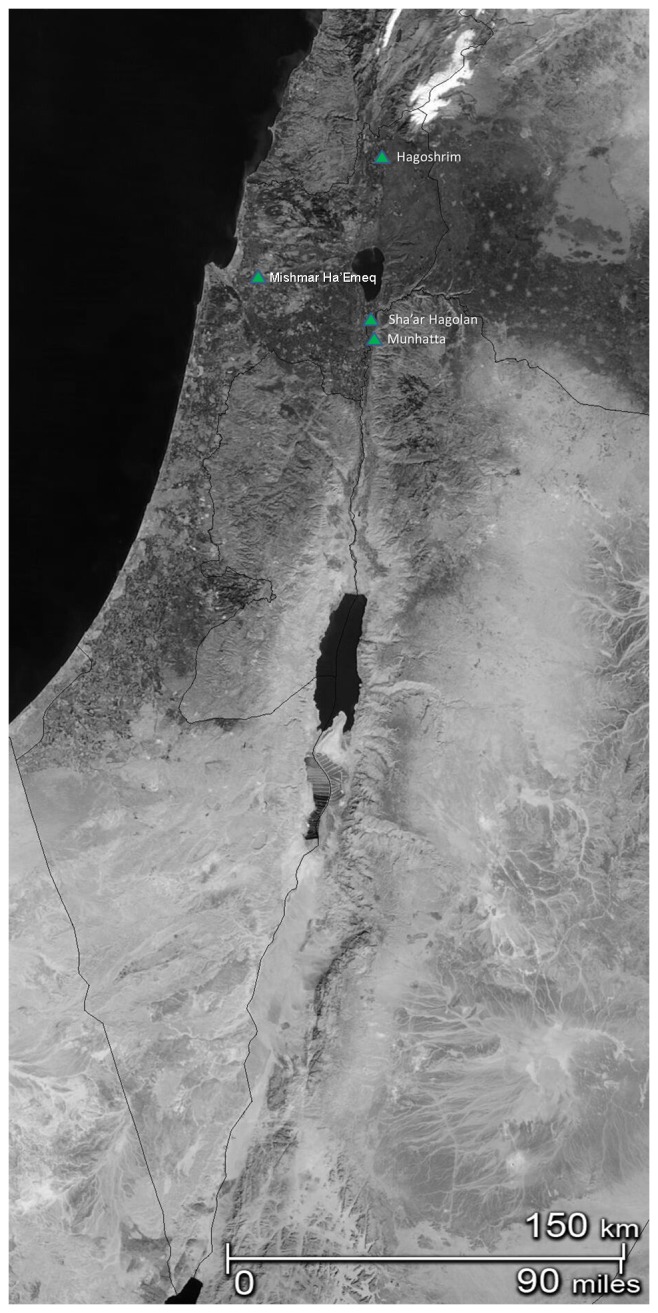
Location map for Sha'ar Hagolan and other sites mentioned in the text.

In this spirit, our study sets out to trace the path towards the domestication of cattle and suids in the southern Levant and, more specifically, in the northern Jordan Valley, Israel, during the late 9^th^ to 8^th^ millennium BP (corresponding with the Pre-Pottery Neolithic C and the Early Pottery Neolithic; henceforward abbreviated as “PPN” and “PN” for Sha'ar Hagolan data). This region, here defined as extending from the confluence of the Rivers Yarmuk and Jordan in the south to the Hula Valley in the North (Figure 1), held in that time period rich riparian and alluvial habitats, which were favorable to wild boar and wild cattle. Because ancient DNA extraction from Neolithic zooarchaeological specimens in the region has so far been unsuccessful [11], we are limited in our inquiry to morphometric and demographic data that are to date the most commonly used methods for documenting the transition from hunting to controlled selection [12].

In the late 9^th^ millennium BP, both suids and cattle undergo a process of diminution in sites across Israel, which registers as a separate event from the reduction in size apparent just following the Holocene climatic amelioration [13]. At roughly the same time, a sharp rise is observed in the frequencies of cattle in the north and central Jordan Valley [14] that indicates a change in human exploitation of these species. Grigson [15] suggested that cattle body-size reduction was preceded by predation on nursery herds, a pattern that is also supported by a high frequency of female cattle. A few centuries later, in the 8^th^ millennium, there is ample and solid (demographic and morphometric) evidence for the fully-domesticated status of cattle [16]. Fully-domesticated pigs are also found in the southern Levant by the early 7^th^ millennium BP. This is evidenced by both demographic and metric data [17]. Earlier, assemblages that are dominated by young suids appear in the region during the 8^th^ millennium [18] together with reduced body-size.

If cattle and pigs are fully-domesticated in the northern Jordan Valley by the early 7^th^ millennium BP, it is reasonable to search for evidence of intensified human exploitation of these animals in the preceding millennium. The zooarchaeological assemblages from the key site of Sha'ar Hagolan fill this gap. Located in the Jordan Valley, south of the Sea of Galilee (Figure 1; for a recent summary see [19] and references therein), the Neolithic village was founded in an alluvial plain, at the confluence of the Rivers Yarmuk and Jordan, in the late 9^th^ millennium BP (Pre-Pottery Neolithic C), reaching its impressive florescence in the early 8^th^ millennium BP (Early Pottery Neolithic Yarmukian culture), under the ameliorated climate of the early Holocene. The excavation of the PN strata at the site revealed a street system, a water well, courtyard houses and large assemblage of portable art, flint and pottery. Faunal and floral investigations conducted at Sha'ar Hagolan show an economy dominated by domesticated caprine herding and founder crop agriculture in the PPN [19]. Evidence for caprine domestication include their dominance in the faunal assemblage from the site (Table 1), their demography and twisted horncore morphology.

**Table 1:** Frequencies of animal bones belonging to different taxa at Sha'ar Hagolan, in number of identified specimens (NISP).

Sample		PPN		PN	
Taxonomy					
Taxon		NISP	%NISP	NISP	%NISP
Caprines	*Capra hircus* and *Ovis aries*	211	44	1011	52
Suids	*Sus scrofa*	91	19	329	17
Cattle	*Bos* sp.	88	18	246	13
Gazelle	*Gazella gazella*	62	13	235	12
Dog	*Canis familiaris*			79	4
Wolf	*Canis lupus*			1	0.1
Equid	*Equus sp.*	10	2	13	1
Roe deer	*Capreolus capreolus*	1	0.2	6	0.3
Fallow deer	*Dama mesopotamica*			2	0.1
Red deer	*Cervus elaphus*	1	0.2		
Tortoise	*Testudo graeca*	1	0.2	8	0.4
Cat	*Felis* sp.	4	1	6	0.3
Hare	*Lepus capensis*			2	0.1
Fox	*Vulpes vulpes*	9	2	1	0.1
Polecat	*Vormela peregusna*	1	0.2		
Total		479	100	1939	100

Diversity			
Index	*Bootstrap P-value, PPNC vs. Yarmukian*	Values	
Richness (S)	0.79	11	13
Shannon E	0.39	0.41	0.31

The alluvial habitats modified by human agricultural activities around Sha'ar Hagolan facilitated encounters between humans and wild cattle and boar. That interaction is important for understanding the observed demographic and biometric changes associated with the domestication of cattle and pigs [20]. We suggest that the social and physiological adaptation of local wild populations to hunting stress were important in their domestication process.

The faunal assemblage of Sha'ar Hagolan consists of two chronostratigraphical phases. The PN phase was radiocarbon dated to 6400–5800 cal. BCE [19], allowing diachronic examination of changes in body-size and demography of cattle and pigs at the eve of their domestication. Thus, it presents a case study for exploring cattle and pig evolutionary trajectories in the southern Levant, at a crucial time for understanding their domestication history in the region.

## Methods

### Ethics statement

All necessary permits were obtained for the described studies. Permission to analyze and published the animal bone assemblage from Sha'ar Hagolan was granted to us by Prof. Yosef Garfinkel, who headed the expedition of the Hebrew University of Jerusalem to that site. Permission to use data from Mishmar Ha'Emeq was granted by Dr. Omry Barzilai and N. Getzov, who directed the excavations at the site on behalf of the Israel Antiquity Authority.

The animal bones from Sha'ar Hagolan presented in this study were recovered using a 2 mm mesh during eleven excavation seasons (1989–1990, 1996–2004). Identification to taxon was carried out using the comparative collection of the Laboratory of Archaeozoology at the University of Haifa. The faunal assemblage from Sha'ar Hagolan is now curated in the Israeli Antiquity Authority storage facilities in Bet-Shemesh, Israel.

Both fused and unfused specimens were measured using Vernier calipers to the nearest 0.1 mm following von den Driesch [21]. The measurements were transformed to a logarithmic ratio between the observed specimen and a reference set. Reference sets included a wild cattle female from Ullerslev, Denmark [15], and an Anatolian wild boar female [22]. It is important to note that the choice of reference sets has no intrinsic importance, and they serve only as benchmarks for comparison between archaeological samples.

The log-transformed measurements were used to compare body-sizes between the PPN and PN suid and cattle bones from Sha'ar Hagolan. We used as a control a sample of wild cattle and boar from the 10^th^ millennium BP (Pre Pottery Neolithic B) site of Mishmar Ha'Emeq in the Jezreel Valley, excavated by Omry Barzilai and Nimrod Getzov of the Israel Antiquity Authority [23]. The bones from Mishmar Ha'Emeq were analyzed by one of the authors (NM) and are currently kept in a Israel Antiquity Authority storage facility in Acre. Although the sample from Mishmar Ha'Emeq is small, it has the merit of being very close in space to Sha'ar Hagolan, preceding the latter by a millennium, but falling safely inside the climatic regime of the early Holocene, and, thus, neutralizing possible biases caused by spatio-temporal body-size variability [13]. Comparison of log-transformed measurements was carried out using One-Way ANOVA, with post-hoc application of Tukey's pairwise comparisons. Suid body-size estimation also relied on a comparison of lower M3 lengths from Sha'ar Hagolan to those of recent wild boar from the Galilee and Golan Heights curated in the Laboratory of Archaeozoology and Paleontology at the Hebrew University in Jerusalem. For comparison we used Student t-test and F tests. Statistical tests were done on PAST 2.10 [24].

Age-at-death was determined by epiphyseal fusion [25] data presented as a survivorship curves. Sex ratios in the archaeological sample were inferred by calculating the second Pearson's skewness coefficient for log-transformed measurements. Positive and negative skewness values were interpreted as female or male dominance in the archaeological assemblages [26].

The interpretation of survivorship curves called for monitoring density-mediated attrition that could differentially delete juvenile bones from the assemblage [27]. Minimum number of elements (MNE) values calculated using a fraction summation approach [28] were also correlated with the use of bone photon-densitometry mineral density values of a sheep [29] for medium- or large-sized mammal bones in each sample. For full details on taphonomic procedures see [30].

## Results

### The Assemblages

The taxonomic composition of the faunal samples from the PPN and the PN of Sha'ar Hagolan are presented in Table 1. The assemblages are both dominated by caprines, followed by suids and cattle. Assemblage diversity is similar between the samples, indicating stability in faunal composition in time. The effect of density-mediated attrition on the compared assemblages is similarly small (Table 2). Fragmentation levels overlap, ranging in value from 0.36 to 0.47 for the medium- and from 0.43–0.50 for large ungulate size-classes. Together, the low and even levels of density-mediated attrition and the similar levels of fragmentation indicate that the earlier and later faunal samples are taphonomically comparable.

**Table 2:** Density-mediated attrition and fragmentation of the Yarmukian and PPNC samples.

	PPN		PN	
	Medium	Large	Medium	Large
**NISP**	530	134	2490	366
**Effect of density on MNE**				
Regression equation	y = −37.141x+29.916	y = −19.033x+11.972	y = −62.866x+70.36	y = −18.196x+14.254
R^2^	0.16	0.21	0.08	0.12
Spearman's r, P	−0.52, 0.09	−0.49, 0.12	−0.34, 0.29	−0.41, 0.19
**Fragmentation (MNE/NISP)**	0.47	0.5	0.36	0.43

### The Cattle

**Figure 2:**
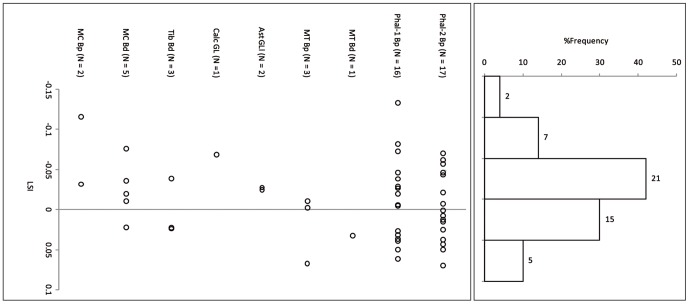
Log-ratio values of Sha'ar Hagolan PPN cattle measurements scaled to the measurements of a reference wild cattle individual [**15**]. The reference specimen is marked by a line on LSI = 0.00.

**Figure 3:**
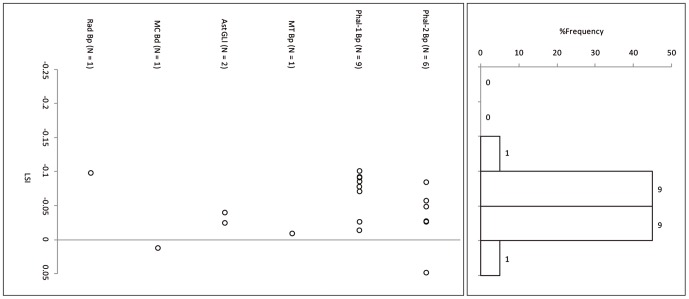
Log-ratio values of Sha'ar Hagolan PN cattle measurements scaled to the measurements of a reference wild cattle individual [**15**]. The reference specimen is marked by a line on LSI = 0.00. Gray fill = unfused bone specimens.

**Figure 4:**
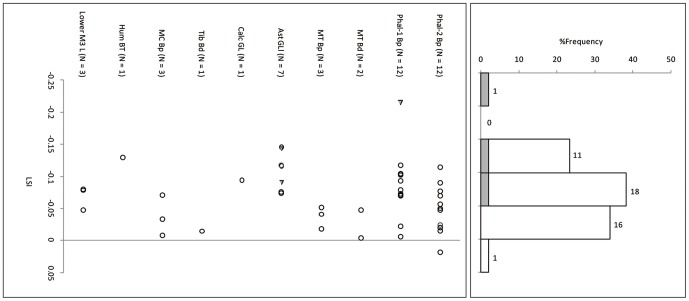
Log-ratio values of Mishmar Ha'Emeq PPNB cattle measurements scaled to the measurements of a reference wild cattle individual [**15**]. The reference specimen is marked by a line on LSI = 0.00. Gray fill = unfused bone specimens.

Cattle body-size at Sha'ar Hagolan is smaller than the reference wild individual (Figures 2 and 3; measurements in Table 3). In the similarly log-transformed reference set from the 10^th^ millennium (Figure 4) the LSI of the archaeological population varies around the reference individual. The body-sizes of the PPN and PN cattle are not statistically different from each other, and both are significantly smaller than the PPNB wild cattle (One-Way ANOVA F = 19.79, P<0.001, Table 4).

**Table 3:** Measurements of cattle bones. Values in italics indicate unfused specimens.

Measurement	Sample	Values (mm)				
Lower M3 L	Sha'ar Hagolan PN	40.8	43.8	40.6		
Hum BT	Sha'ar Hagolan PN	66.1				
Rad Bp	Sha'ar Hagolan PPN	79.7				
MC Bp	Sha'ar Hagolan PN	68.6	63	72.7		
	Mishmar Ha'Emeq PPNB	68.8	56.7			
MC Bd	Sha'ar Hagolan PPN	75.2				
	Mishmar Ha'Emeq PPNB	67.2	76.9	71.3	69.8	61.3
Tib Bd	Sha'ar Hagolan PN	75.5				
	Mishmar Ha'Emeq PPNB	82.2	82.5	71.4		
Calc GL	Sha'ar Hagolan PN	133				
	Mishmar Ha'Emeq PPNB	140.9				
Ast GLl	Sha'ar Hagolan PN	63.6	59.4	***59.8***	63.4	***67.5***
		70.1	69.7			
	Sha'ar Hagolan PPN	78.4	75.8			
	Mishmar Ha'Emeq PPNB	77.9	78.5			
MT Bp	Sha'ar Hagolan PN	59.5	56.5	55.1		
	Sha'ar Hagolan PPN	60.7				
	Mishmar Ha'Emeq PPNB	61.7	60.6	72.4		
MT Bd	Sha'ar Hagolan PN	61	67.5			
	Mishmar Ha'Emeq PPNB	73.4				
Phal-1 Bp	Sha'ar Hagolan PN	30.7	33.2	29.8	30.9	38.5
		31.5	32.5	33.2	***23.8***	37.1
		33	30.8			
	Sha'ar Hagolan PPN	37.8	32.6	32	33.1	31.6
		31.5	31.6	36.7	30.9	
	Mishmar Ha'Emeq PPNB	41.5	36.7	44.9	38.5	37.3
		28.7	43.8	36.5	33	35.1
		42.7	35.7	42.5	38.6	42
		32.3				
Phal-2 Bp	Sha'ar Hagolan PN	37.6	34.8	30.2	34.1	32.1
		34.4	27.7	31.6	32.3	30.7
		29.3	30.7	30.9	32.8	29.5
		29.9	26.2			
	Sha'ar Hagolan PPN	29.6	32.2	31.6	33.9	40.3
		33.8				
	Mishmar Ha'Emeq PPNB	30.6	38.2	32.4	42.3	40.4
		34.3	39.8	36.7	37.1	39.3
		37.3	32.6	35.4	31.2	31.6
		36.1	32.2			

**Table 4:** One-Way ANOVA results and post-hoc tests for the comparison of log-ratio values of cattle measurements from PPNB Mishmar HaEmeq, and PPN and PN Sha'ar Hagolan.

One-Way ANOVA: F = 19.79, P = 0.0001			
Tukey's Pairwise comparisons (Q\p)			
	PN	PPN	Mishmar HaEmeq
PN	0	0.2018	*0.000116*
PPN	2.434	0	*0.002553*
Mishmar HaEmeq	7.28	4.845	0

**Figure 5:**
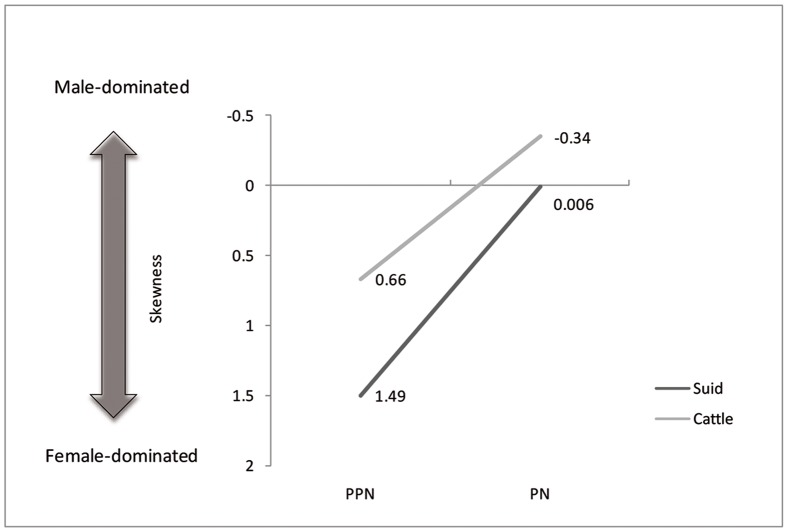
The shift in Pearson's skewness for cattle and suid log-ratio values between the PPN and PN at Sha'ar Hagolan.

In the earlier period (PPN), cattle measurements are positively skewed (Pearson's skewness = 0.66), indicating that most values cluster at the lower half of the range and that, therefore, we likely observe a female-dominated sample (Figure 5). In contrast, the later sample is negatively skewed (Pearson's skewness = −0.33), indicating that most values cluster at the upper half of the range and that, therefore, we observe a male-dominated sample.

**Figure 6:**
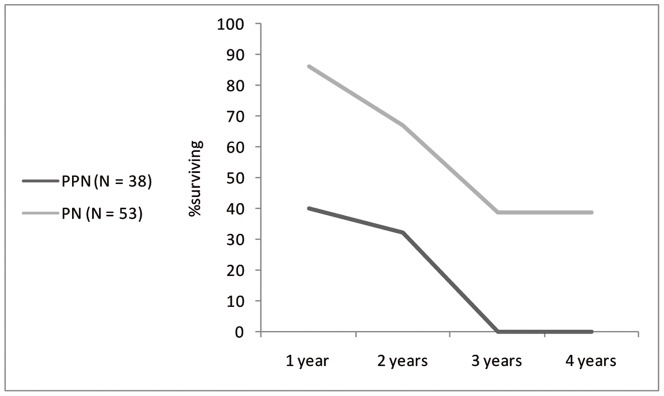
Survivorship curve for Sha'ar Hagolan cattle population of the PN and PPN, based on epiphyseal fusion data in Supplement 3.

The survivorship curve of PPN cattle is far steeper than the PN, to such an extent that very few mature animals appear to be present (Table 5, Figure 6). The PN survivorship curve from Sha'ar Hagolan shows a slight mortality in the first year of life, with a large part of the population surviving to maturity (at least four years at death).

**Table 5:** Epiphyseal fusion data for cattle from Sha'ar Hagolan.

		PPN		PN	
Element	Age (years)	Fused	Unfused	Fused	Unfused
Acetabulum	1	1	3	1	1
Scapula	1	1		5	
*TOTAL*	*1 year*	*2*	*3*	*6*	*1*
Humerus, distal	1.5		3	1	4
Radius, proximal	1.5	1			
Phalanx 1	1.5	11		11	2
Phalanx 2	1.5	9	2	13	1
*TOTAL*	*2 years*	*21*	*5*	*25*	*7*
Metacarpus, distal	2.5		3	3	
Tibia, distal	2.5		1	1	1
Calcaneum	3			1	4
Metatarsus, distal	3		1	2	
*TOTAL*	*3 years*	*0*	*5*	*7*	*5*
Femur, proximal	3.5				
Femur, distal	3.5			1	
Humerus, proximal	4				
Radius, distal	4		1	1	
Ulna	4		1		
Tibia, proximal	4				
*TOTAL*	*4 years*	*0*	*2*	*2*	*0*

### The Suids

**Figure 7:**
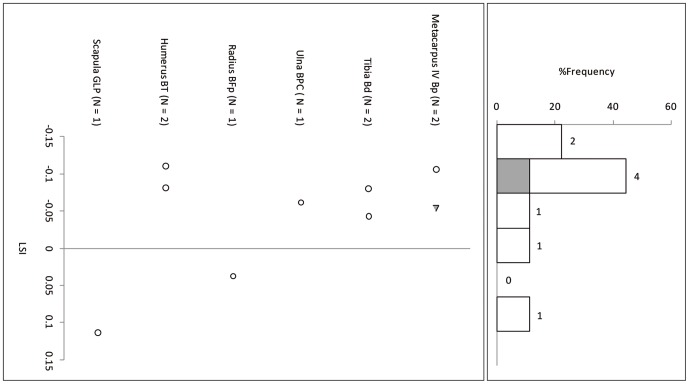
Log-ratio values of Sha'ar Hagolan PPN suid measurements scaled to the measurements of a reference wild boar female individual [**22**]. The reference specimen is marked by a line on LSI = 0.00. Gray fill = unfused bone specimens.

**Figure 8:**
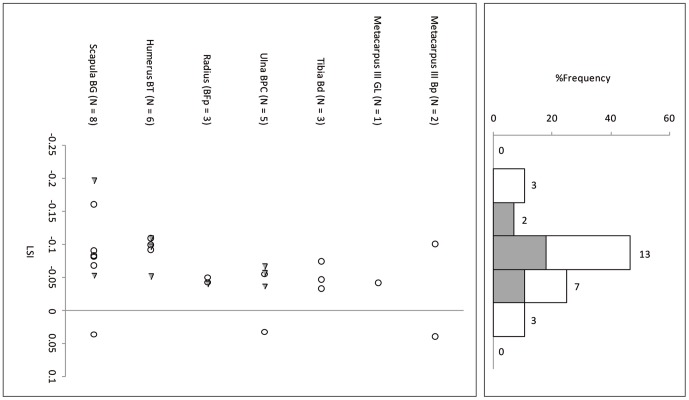
Log-ratio values of Sha'ar Hagolan PN suid measurements scaled to the measurements of a reference wild boar female individual [**22**]. The reference specimen is marked by a line on LSI = 0.00. Gray fill = unfused bone specimens.

**Figure 9:**
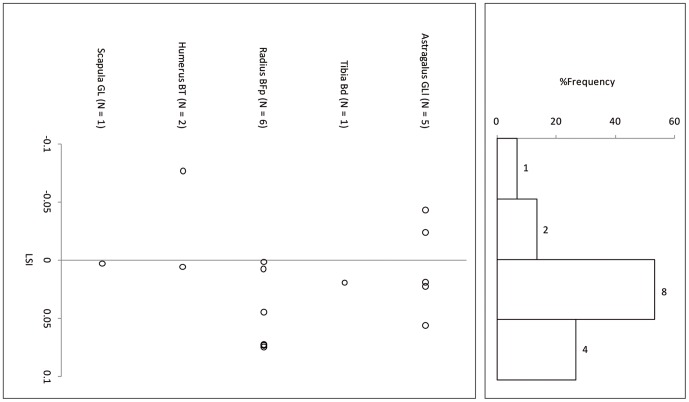
Log-ratio values of Mishmar Ha'Emeq PPNB wild boar measurements scaled to the measurements of a reference wild boar female individual [**22**]. The reference specimen is marked by a line on LSI = 0.00. Gray fill = unfused bone specimens.

Suid log-transformed post-cranial measurements (Table 6) follow the cattle pattern in being similarly smaller than the standard animal, itself a small female, and in being statistically indistinguishable from each other but significantly smaller than a co-regional 10^th^ millennium BP population (Figures 7, 8 and 9, Table 7). A comparison of lower M3 length measurements to those of a recent sample of wild boar from northern Israel (Table 8) shows the PN sample from Sha'ar Hagolan to have been significantly smaller than this recent sample (t = 2.31, P = 0.03). The earlier sample shows strong positive skewness (Pearson's skewness = 1.50; Figure 6) indicating a sample dominated by females. An increase in the number of larger individuals, very likely male animals, occurs in the PN (Pearson's skewness = 0.01).

**Table 6:** Measurements of suid bones. Values in italics indicate unfused specimens.

Measurement	Sample	Value (mm)				
Scapula GLP	Sha'ar Hagolan PPN	49.9				
Scapula BG	Sha'ar Hagolan PN	22.8	22.3	***24.4***	***17.5***	19
		23.5	29.9	22.7		
Scapula GL	Mishmar Ha'Emeq PPNB	32.7				
Humerus BT	Sha'ar Hagolan PPN	31.2	29.1			
	Sha'ar Hagolan PN	***29.1***	29.2	29.8	***33.3***	***30***
		30.3				
	Mishmar Ha'Emeq PPNB	31.4	38			
Radius BFp	Sha'ar Hagolan PPN	37.3				
	Sha'ar Hagolan PN		***31.2***	31	30.5	
	Mishmar Ha'Emeq PPNB	40.6	34.8	37.9	40.4	40.5
		34.3				
Ulna BPC	Sha'ar Hagolan PPN	22				
	Sha'ar Hagolan PN	***21.7***	27.3	***22.3***	22.2	***23.3***
Tibia Bd	Sha'ar Hagolan PPN	27.9	30.4			
	Sha'ar Hagolan PN	31.1	28.2	30.1		
	Mishmar Ha'Emeq PPNB	35				
Astragalus GLl	Mishmar Ha'Emeq PPNB	50	43	49.6	54	44.9
Metacarpus IV Bp	Sha'ar Hagolan PPN	15	***16.9***			
Metacarpus III GL	Sha'ar Hagolan PN	78.9				
Metacarpus III Bp	Sha'ar Hagolan PN	16.4	22.7			

**Table 7:** One-Way ANOVA results and post-hoc tests for the comparison of log-ratio values of PPNB, PPN and PN suid measurements from Sha'ar Hagolan and Mishmar HaEmeq.

One-Way ANOVA F = 11.49, P<0.001			
Tukey's Pairwise comparisons (Q\p)			
	PPNB	PPN	PN
PPNB	0	*0.01572*	*0.000607*
PPN	4.087	0	0.4701
PN	5.758	1.671	0

**Table 8:** Measurements of suid lower third molars from Sha'ar Hagolan and of recent specimens from the region.

Recent				Sha'ar Hagolan	
Catalogue Number		Sex	Lower M3 Length	Context	Lower M3 Length
M3964		Male	38.2	PN	42.5
M3958		Male	40.4	PN	35.2
H6985			38.1	PN	37.8
M8189		Female	39.6	PN	38.4
M3962		Male	38.2	PN	32.5
M6617		Male	36.1	PN	37.4
M3961			39	PN	34.9
M3957		Female	39.8	PPN	34.8
M3959		Female	40.2		
M6170		Male	39.2		
M3960		Male	39.9		
M8167			37.3		
M8153			39.9		
M8175			38		
M8165			37.3		
M8175			37.8		
N:	16			N:	8
Mean:	38.68			Mean:	36.68
95%:	(38.03 39.35)			95%:	(34.14 39.23)
Variance:	1.55			Variance:	9.28
F:	5.99			p(same):	0.003
t:	2.31			p(same):	0.03

**Figure 10:**
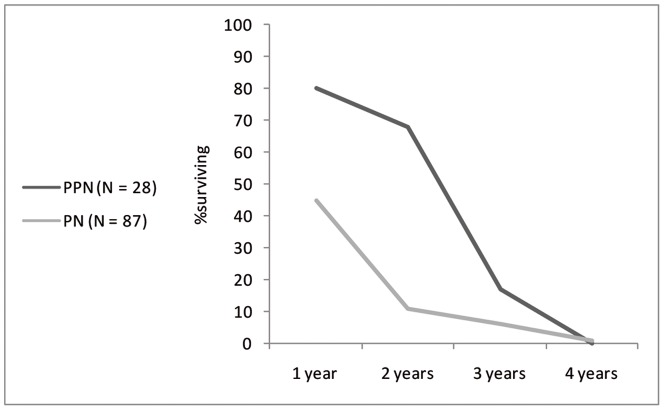
Survivorship curve for Sha'ar Hagolan suid population of the PPN and PN, based on epiphyseal fusion data in **Table 9**.

Suid survivorship curves (Table 9, Figure 10) indicate intensive culling in both earlier and later samples. A culling peak is apparent at the age of three years in the PPN and at the first two years of life in the later sample.

**Table 9:** Epiphyseal fusion data for suids from Sha'ar Hagolan.

		PPN		PN	
Element	Age (years)	Fused	Unfused	Fused	Unfused
Acetabulum	1	1		3	
Scapula	1	2		5	8
Radius, proximal	1	1	1	1	1
Phalanx 2	1	6		7	4
Humerus, distal	1	2	2	2	9
TOTAL	1 year	12	3	18	22
Phalanx 1	2	3		2	10
Tibia, distal	2	2		2	4
Metacarpus	2	1	1	1	1
TOTAL	2 years	6	1	5	15
Metatarsus	2.5			1	2
Calcaneum	2.5	1	3	5	3
TOTAL	3 years	1	3	6	5
Femur, proximal	3.5				1
Ulna, proximal	3.5		1		6
Femur, distal	3.5				3
Radius, distal	3.5		1	1	3
Humerus, proximal	3.5				
Tibia, proximal	3.5			1	1
TOTAL	4 years	0	2	2	14

### Synoptic View of Survivorship

**Figure 11:**
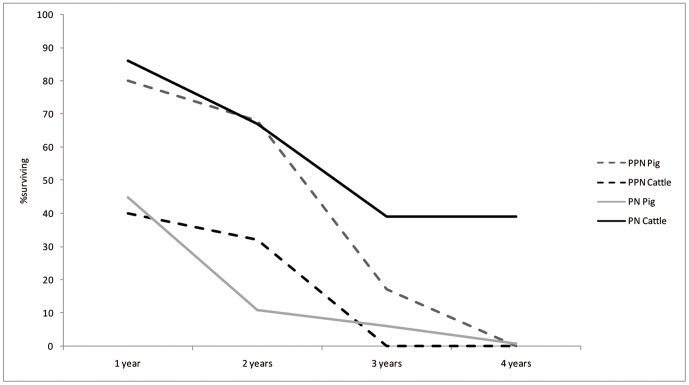
Survivorship curves for Sha'ar Hagolan cattle and suids. Note that with the transition from the PPN (dashed lines) to the PN (solid line) the curves diverge to patterns that are reminiscent of known husbandry survivorship patterns.

A synoptic look at the survivorship curves of both cattle and suids from PPN and PN Sha'ar Hagolan is presented in Figure 11. It cannot be demonstrated statistically, due to sample size limitations, that cattle and suid survivorship rates changes between the PPN and the PN at the species level. Nevertheless, the graph also indicates similarity between the curves representing PPN survivorship and its dissolution in the PN into two very distinct curves—one for the suids and one for cattle. This observation can be quantified by conducting a rarified Chi-squared analysis per age class (Table 10), which demonstrates that the mortality profiles are dissimilar for PN cattle and suids for all age classes except the first year, while such dissimilarity cannot be shown for the PPN data. The convergent *versus* divergent nature of mortality can be thus demonstrated. The convergent PPN survivorship pattern cannot be explained by any management practice known to the authors, while the divergent pattern is expected for the husbandry of both cattle and pigs.

**Table 10:** Rarified Chi-squared analysis of PN and PPN survivorship of cattle and suids at Sha'ar Hagolan.

	PPN					
	Cattle		Pigs			
Year	Fused	Unfused	Fused	Unfused	Chi-squared	P(same)
4	23	15	19	9	0.374	0.829
3	23	13	19	7	0.583	0.747
2	23	8	18	4	0.427	0.808
1	2	3	12	3	N/A	N/A

	PN					
	Cattle		Pigs			
Year	Fused	Unfused	Fused	Unfused	Chi-squared	P(same)
4	40	13	31	56	20.914	0.000
3	38	13	29	42	13.586	0.001
2	31	8	23	37	16.146	0.000
1	6	1	18	22	N/A	N/A

The Chi-squared values and significance in the rightmost columns refer to the 2×2 cross-tabulation of cattle and suid fused and unfused skeletal elements appearing in the central columns, arranged by period (PPN, top; PN, bottom). The “fused” and “unfused” frequencies in the central columns are cumulative and include all the specimens that fuse by the age listed in the leftmost column. The decomposition of the survivorship rates into this rarified Chi-squared design demonstrates that there is clear dissimilarity between cattle and pig survivorship in the PN. Dissimilarity cannot be demonstrated for PPN survivorship.

The demographic and metric data for PPN and PN suids and cattle are based on relatively small sample sizes. These small samples represent all the measureable specimens from Sha'ar Hagolan that are or will be available for analysis, since the excavation of the site will not be renewed in the foreseeable future. Statistical significance, which takes into account sample-size limitations, is demonstrated for the key variables of a diachronic shift in body-size and changes in mortality profiles. The data presented above are therefore felt to be sufficient for hypothesizing on the pathway to domestication in the study region. These hypotheses, however, will have to stand more rigorous testing in the future.

## Discussion

The results of the metric and demographic analyses of cattle and suids from Sha'ar Hagolan are important for tracking the initial stages of suid and cattle domestication in the Jordan Valley, with broader consequences for understanding the timing and domestication trajectories of these taxa.

Body-size in cattle and suids was significantly smaller than that of the reference specimens and of local wild populations from the earliest phase of settlement at the site, in the PPN. The small body-size is connected with female-dominated sex-ratio for both taxa. Both cattle and suids in that period share similar steep survivorship curves, with the culling peak in the younger-adult ages (three years). Survival to later adulthood appears to have been a rare occurrence in PPN populations. This is apparently not the result of a conservative herding strategy for either cattle or pigs, if we also take into account the female-dominated sex ratio, since it means very early culling of the reproductive core of the herd [31].

In the PN, the steep cattle mortality curve changes drastically in the direction of delayed culling: nearly half the population survives to older age. Also notable is the change in male-dominated sex ratio. This observation is in line with the measurements of unfused specimens, which are not large in relation to the cattle population average and preclude selective culling of young males. The small population of animals that were retained to an older age—in marked contrast with that reflected in the PPN sample—speaks for the presence of fully-domesticated cattle. Pig demography in the PN also demonstrates a sharp break with the previous millennium's culling practices. For pigs, the transition to the PN meant a younger age-at-death, with a sharp culling peak at the first and second years of life. The sharp positive skew in the PPN metric data, interpreted in terms of female dominance, disappears. In ordinal terms, more males are present. This very young age-at-death for pigs and the larger number of males present in the assemblage argues in favor of the presence of domesticated suids in the PN.

When combined with the evidence from the two sites where documentation of demography exists for the time period in the study region [17], [18], there appears to be a regional change in suid utilization patterns in the early 8^th^ millennium followed a few centuries later by similar events further to the west in the Carmel region [32].

The existence of domesticated cattle during the 8^th^ millennium of the southern Levant is a solidly-based hypothesis [15]. Our observations on the full morphological and demographic domestication of suids and cattle in 8^th^ millennium Sha'ar Hagolan are in accord with the south Levantine context. It is also apparent that the transition to the PN at the site saw a sharp change in the mortality profiles of both taxa towards well-documented patterns for fully-domesticated animals, which do not match the steep mortality curves of the PPN at the site.

Whereas the conclusions on the domesticated state of cattle and suids in the PN settlement of Sha'ar Hagolan accord well with the expectations and data of previous research, which relied on shifts in taxonomic frequencies and metric data [13]–[15], we are left to interpret the record pertaining to the game-management strategy in the preceding PPN. This record captures the penultimate stage of domestication at Sha'ar Hagolan. We tend to view the steep mortality profiles combined with a reduction in body-size and female dominance, occurring simultaneously and similarly in both cattle and suids, as reflecting the demographic structure and body-size changes of a population under severe hunting stress combined with habitat depletion [26].

One of the consequences of overhunting is a reduction in mean body-size that can be observed in faunal assemblages [33]–[39]. Body size-reduction can results from (a) preferential targeting of larger prey individuals, mainly mature males; (b) generally higher recruitment rate in the population caused by reduced intra-taxon competition; (c) lower chances of survival to maturity; and, importantly, (d) selection for early sexual maturation that inhibits somatic growth [40], [41].

Overhunted ungulate populations will also display steep mortality profiles, with high juvenile attrition—the result of high recruitment rate brought about by reduced intra-taxon competition and lower chances of survival to maturity. Such populations are expected to show higher female frequency due to the general human preference to hunt the larger male animals [42] either for cost-effectiveness in foraging [43], prestige [44], or as a strategy for resource management [45]. A juvenile and female-dominated population structure should result in the reduction in mean population body-size, a process exacerbated by viability selection for early sexual maturation that inhibits somatic growth [39], [46]. The reduction in body-size combined with steep mortality profiles may indicate overhunting combined with reduced ecological carrying capacity in archaeozoological assemblages [26].

During the 10^th^ and 9^th^ millennia BP, intensive hunting of wild cattle and wild boar was practiced in the northern Jordan Valley, departing from the emphasis on gazelles and deer observed in the preceding hunting cultures of the region [47]. This change occurred when the first plant agriculturalists in the Neolithic of the southern Levant increased their settlement of alluvial settings, which are favorable for plant cultivation in terms of water and soil availability, as part of the great shift towards agricultural economy [3]. Expansion into alluvial habitats and their agricultural modification resulted in hunting pressure on ungulate taxa that thrived in alluvial fans—wild cattle [48] and wild boar [49]. Susceptibility to overhunting was possibly the result of higher encounter rates caused by co-habitation and of crop-raiding [20], [50]. This hypothesis agrees with the observation made by Grigson [15] on the targeting of wild cattle nursery herds at that time period, an observation that may also explain the dominance of female animals in the archaeological assemblage.

From an optimal-foraging perspective, if the body-size of the hunted cattle and pigs was reduced to the point where the adult female body-size was incorporated in the diet – i.e., when preferential hunting of males ceases, adult females will become more dominant in the diet. This preference is especially true if very few territorial adult males remained and the majority of the population is aggregated in nursery herds composed of adult females and young males and females. Since the measurements from PPN Sha'ar Hagolan do not include young individuals, an observed pattern of female domination emerges. This pattern was first noted and interpreted by Grigson [15: 98]: “However although the cattle of the seventh millennium bc sites were of wild size, there is a suggestion that in the western part of the area there was a stress on females; this could be interpreted as predation upon nursery herds and possibly as a first step towards domestication”.

Both overhunting and domestication manifest in the metric data in a similar way, by population mean body-size reduction and by a low proportion of adult males. Therefore, it is pertinent to explore the consequences of overhunting as possible candidates for shaping the archaeozoological signature of domestication. Further, overhunting may have caused domestication, as both motivation and an unconscious selective mechanism that pre-adapted prospective domesticates to full-fledged husbandry. Reduced body-size, sexual dimorphism, and enhanced fertility, when combined with selection against aggressively territorial individuals (males) are pre-adaptations to domestication caused by a simple, non-conscious process [51] on the part of humans: overhunting. But how does overhunting lead to domestication?

Alvard and Kuznar [52] suggest that overhunting is typical of human societies [53], and that measures of conservation are taken when the risk in deferring a decision to hunt an encountered animal is small in relation to the potential future benefit. Strategies of conservation, when applied, often appear along with intensified territorialism [54], [55], which is necessary to assure the conservator that he will be the one to enjoy the conserved resource at a future time [56]. There is a smooth transition from staking out hunting territories, in which small populations of young- and female-dominated animals are found, to husbandry (Figure 11)—especially in a context where herd management was already practiced along with the necessary techniques for limiting animal movements. This view of conservation of overhunted animal species as a trajectory towards domestication, in a social context of increased territoriality and ownership of resources [57], appeals directly to the interpretation of the data from the northern Jordan Valley.

**Figure 12:**
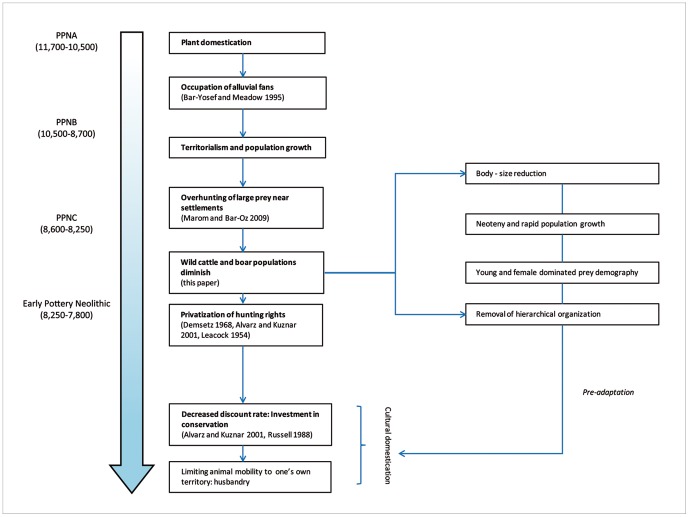
Possible path to the domestication of cattle and pigs at Sha'ar Hagolan.

Under this scenario (Figure 12), the shift towards sustainable population management followed the reduction in body-size caused by over-hunting, which in and by itself was pre-adaptive to domestication by selecting for high population turnover rate, younger birth age, smaller body-size and neoteny; and this selection could have been complemented by the elimination of large and aggressive individuals, which allowed humans to take their role as leaders in the hierarchical structure of the ungulate group [58]. The overhunting hypothesis may therefore be viewed as one of the animal management practices preceding complete domestication. It is marked, however, by low adult survivorship, which precludes the safeguarding of a reproductive herd core. Similar, although not identical, processes of intensification seem to have marked other sites in the study region [15], [17], [18].

The possibility of an ‘overhunting’ trajectory to domestication affects the interpretation of demographic and metric data as markers for domestication. It would suggest that in some cases body-size reduction could precede intentional demographic management of herd animals, and may be the result of a non-intentional process that was pre-adaptive to it—as suggested some years ago by Tchernov and Kolska-Horwitz [51]. Hopefully, future work in the study region will allow further consideration of these ideas, which should at their present state be regarded as hypotheses.
